# Supplementary material for: Whole genome structural predictions reveal hidden diversity in putative oxidative enzymes of the lignocellulose-degrading ascomycete Parascedosporium putredinis NO1
Source: Microbiol Spectr. 2023 Oct 9;11(6):e01035-23. doi: 10.1128/spectrum.01035-23 (PMC10714830; doi:10.1128/spectrum.01035-23)
Supplement: Legends of supplemental files and tables — Legends of supplemental files 1 and 2 and Tables S1 to S3. [file spectrum.01035-23-s0003.docx]

**Legends of supplemental files and tables**

**Supplemental file 1.** Gene expression of interesting sequences. Sequences identified solely by structural searching approaches were considered interesting and were searched for in transcriptomic data from triplicate cultures of *P. putredinis* NO1 grown on glucose or grown on wheat straw with samples taken at days 2, 4, and 10. Gene expression values are presented in transcripts per million (TPM).

**Supplemental file 2.** Ascomycete genome annotations. Annotations of all ascomycete genomes used in this analysis (n = 2570).

**Table S1.** Coding regions of LPMO related proteins identified through genome searching approaches with the sequence of an *A. niger* AA9 LPMO (E-value cut-off = 1 x 10^-5^), the Pfam AA9 HMM (Significance threshold = 0.01), and the structure of the *A. niger* AA9 LPMO (Lowest percentage match = 50%).

**Table S2.** Coding regions of laccase related proteins identified through genome searching approaches with the sequence of an *A. niger* AA1 laccase (E-value cut-off = 1 x 10^-5^), the bespoke laccase and multicopper oxidase HMM constructed from sequences from the laccase engineering database (Significance threshold = 0.01), and the structure of the *A. niger* AA1 laccase (Lowest percentage match = 30%).

**Table S3.** Coding regions of peroxidase related proteins identified through genome searching approaches with the sequences of an MnP from *A. subglaciale*, LiP from *F. oxysporum*, and VP from *P. confluens* (E-value cut-off = 1 x 10^-5^), the bespoke peroxidase HMM constructed from MnP, LiP, and VP seqeunces in the fPoxDB database (Significance threshold = 0.01), and the structure of the same three peroxidases used for sequence searches (Lowest percentage match = 30%).
