# Supplementary figures and images for: Whole genome structural predictions reveal hidden diversity in putative oxidative enzymes of the lignocellulose-degrading ascomycete Parascedosporium putredinis NO1
Source: Microbiol Spectr. 2023 Oct 9;11(6):e01035-23. doi: 10.1128/spectrum.01035-23 (PMC10714830; doi:10.1128/spectrum.01035-23)

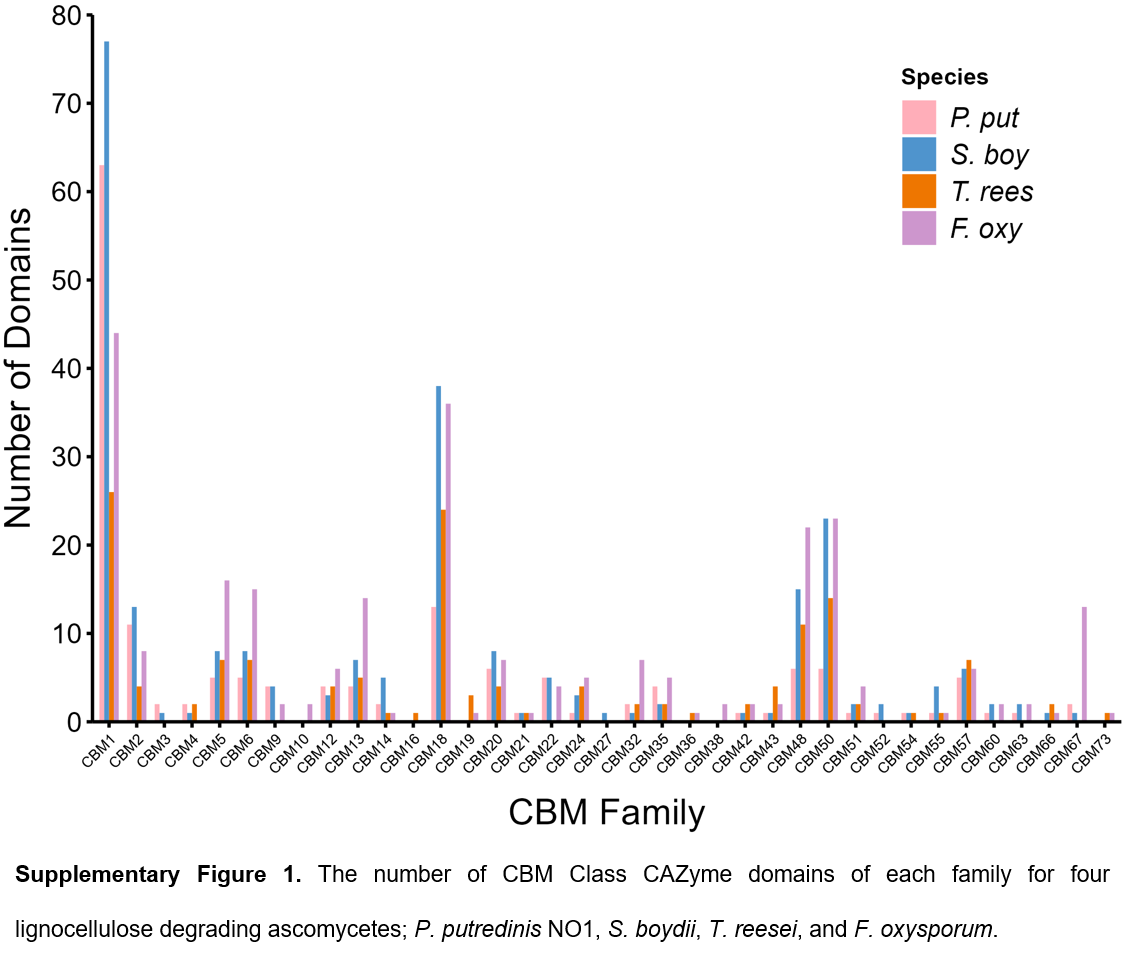

Supplement: Supplemental Figure S1 — , visualizing the CBM family allocations across four asocmycetes. [file spectrum.01035-23-s0007.tif]

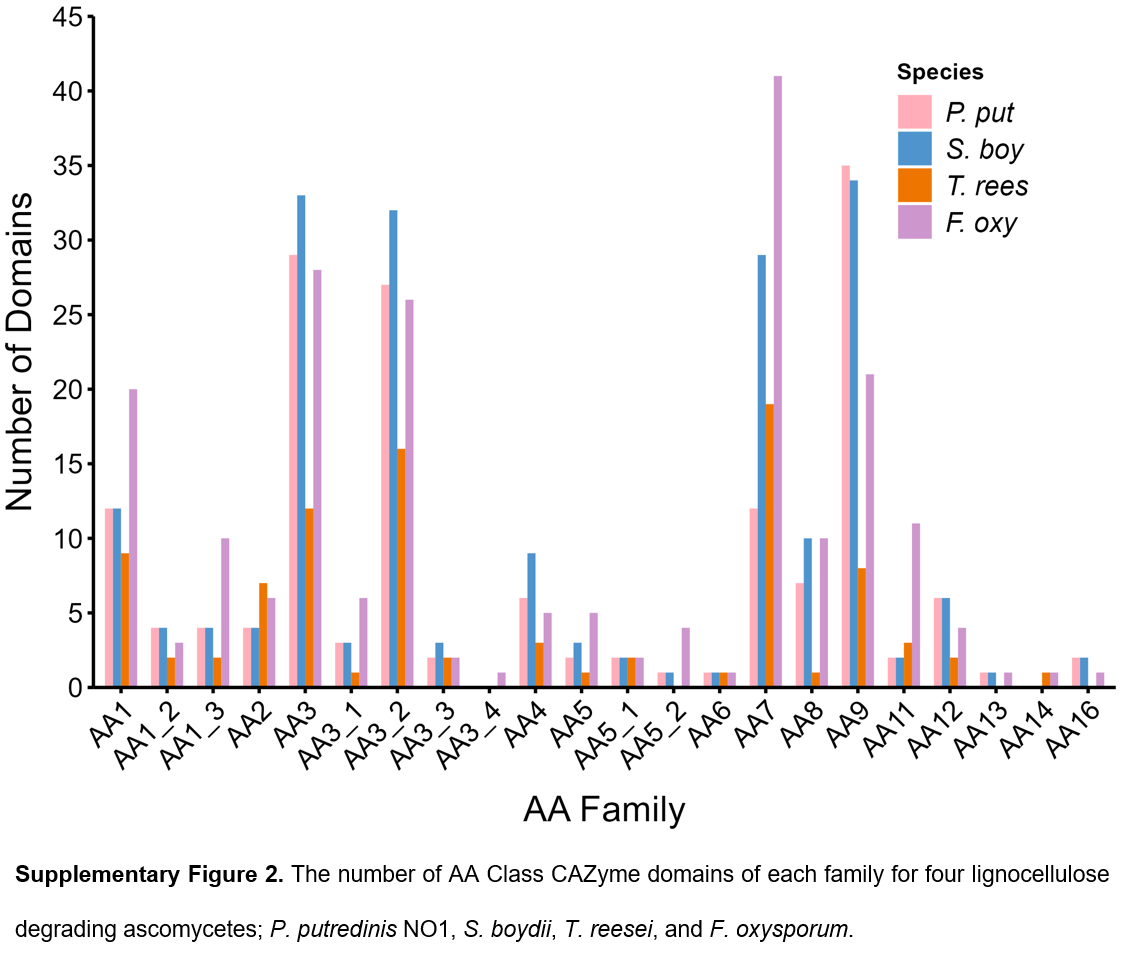

Supplement: Supplemental Figure S2 — , visualizing the AA family allocations across four ascomycetes. [file spectrum.01035-23-s0008.tif]

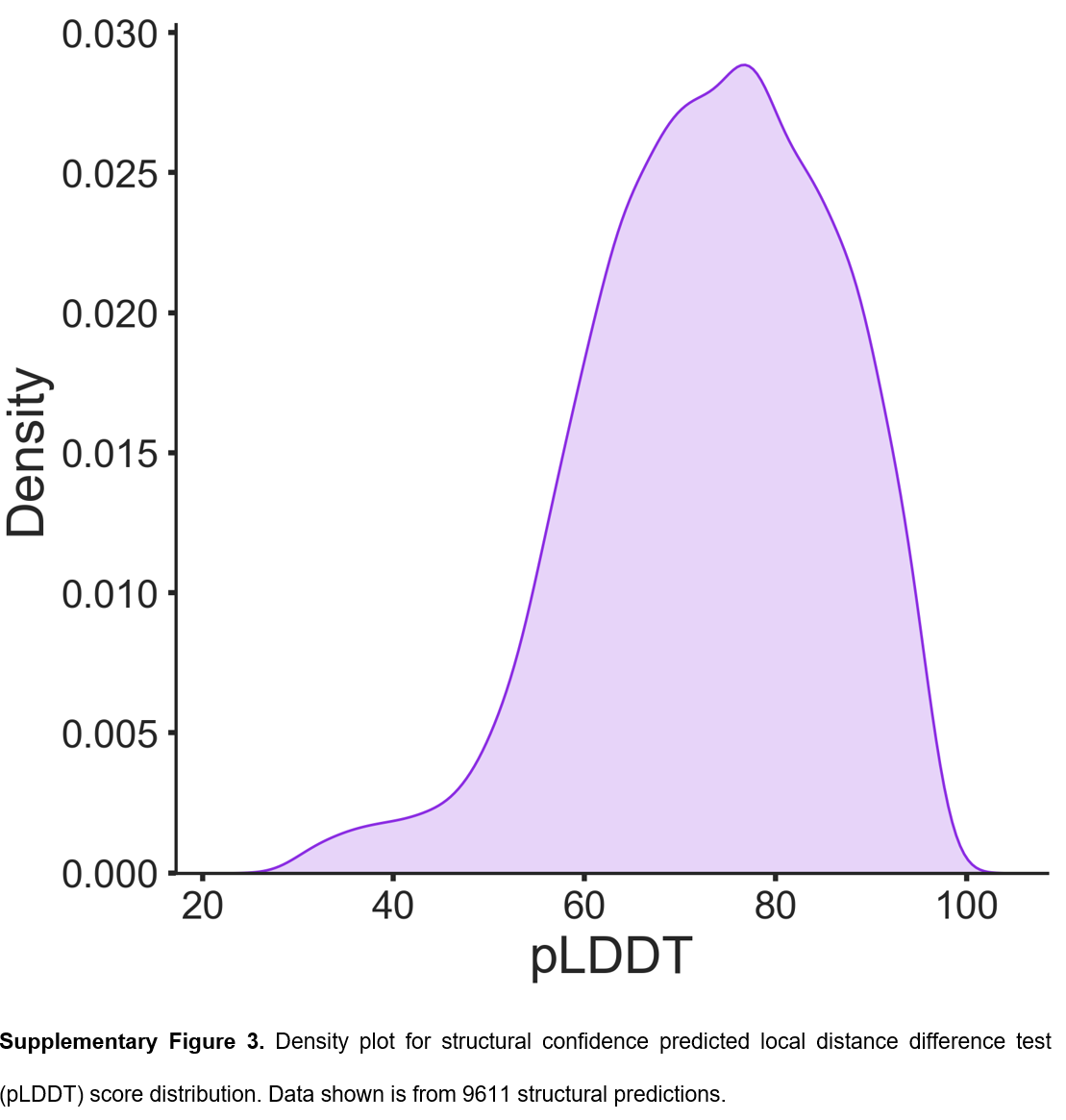

Supplement: Supplemental Figure S3 — , vizualizing pLDDT score distribution for genome predicted structures. [file spectrum.01035-23-s0009.tif]
